# UV-Vis spectroscopy in the characterization and applications of smart microgels and metal nanoparticle-decorated smart microgels: a critical review

**DOI:** 10.1039/d4ra07643e

**Published:** 2024-11-29

**Authors:** Muhammad Arif, Hamid Raza, Toheed Akhter

**Affiliations:** a Department of Chemistry, School of Science, University of Management and Technology Lahore 54770 Pakistan Muhammadarif2861@yahoo.com Muhammadarif@umt.edu.pk; b Department of Chemical and Biological Engineering, Gachon University Seongnam-13120 Republic of Korea toheed@gachon.ac.kr

## Abstract

Smart microgels (SMGs) and metal nanoparticles (MNPs)-decorated smart microgels have garnered significant attention because of their responsive behavior. Both SMGs and their hybrids are extensively used in adsorption, drug delivery, and catalysis, which can be characterized with expensive instruments. UV-Vis spectroscopy is a technique that can easily monitor these processes, and it can also identify the presence of noble metal nanoparticles, like Ag and Au, in smart microgels with the help of the surface plasmon resonance wavelength of these metals. This technique is widely employed to determine the size and shape of MNPs present in microgels. Additionally, UV-Vis spectroscopy enables the investigation of optical behaviors of noble metal nanoparticles under various stimuli. Time-dependent UV-Vis spectroscopy can monitor the kinetics of swelling and deswelling in MGs and HMGs. It also facilitates the study of metal nanoparticle growth within polymer networks or the growth of polymer networks around metal nanoparticle cores. Furthermore, UV-Vis spectroscopy can be used to explore numerous applications of microgels and hybrid substances in photonics, sensing, catalysis, and adsorption. This tutorial review highlights the use of UV-Vis spectroscopy in the characterization and applications of both smart microgels and their hybrids in adsorption and catalysis, considering the recent research advancements in these fields.

## Abstract 2

Smart microgels (SMGs) and metal nanoparticles (MNPs)-decorated smart microgels have garnered significant attention because of their responsive behavior.

## Graphical abstract

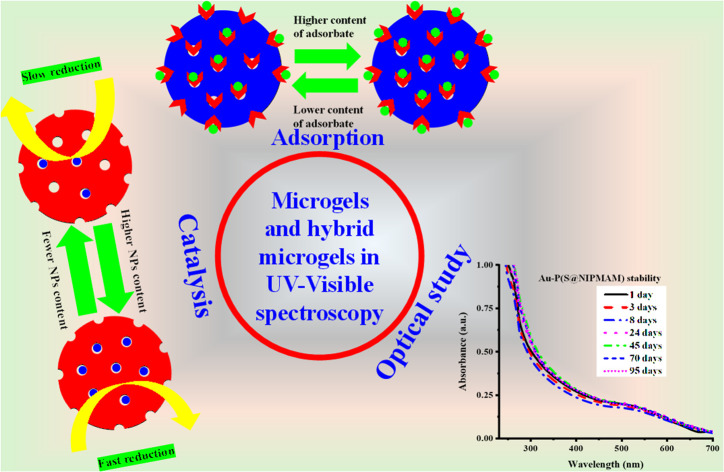

## 1. Introduction

The synthesis and characterization of metal nanoparticles for different applications are active research areas in these days.^1–5^ Strategies for their fabrication and characterization are continually improving.^6–11^ Controlling the shape and size of nanoparticles is crucial for their applications across different fields.^12–14^ However, the instability of these nanoparticles is a challenge that hinders their potential. The reason behind this instability is their high surface energy, leading to aggregation and conversion into massive products. To stabilize metal nanoparticles, various agents such as microgels,^15–18^ block copolymers,^19^ dendrimers,^20^ and surfactants^21^ are used. Metal nanoparticles fabricated with microgels exhibit the properties of both polymer microgels and nanoparticles, forming hybrid microgels (HMGs).^22^ These HMGs (metal nanoparticle-fabricated microgels) have potential applications in optics,^23^ electronics,^24^ catalysis,^25,26^ photonics,^27^ and biomedicine.^28^ HMGs are characterized using techniques, such as scanning electron microscopy (SEM),^29^ atomic force microscopy (AFM),^30^ dynamic light scattering (DLS), X-ray diffraction (XRD), Fourier transform infrared spectroscopy (FTIR),^31^ transmission electron microscopy (TEM),^32^ and ultraviolet-visible (UV-Vis) spectroscopy.^33^ These techniques have their own advantages, making it difficult to directly compare them since each one serves a specific purpose and provides unique information. All these techniques provide only one specific information related to the samples, whereas UV-Vis spectroscopy provides a lot of information related to the characterization as well as applications of microgels and hybrid microgels. It is unique among the mentioned techniques in its ability to investigate the adsorption of pollutants with both microgels^34^ and HMGs^35^ and the kinetics of deswelling and swelling in HMGs^5^ and polymer microgels.^36^ It is commonly used for characterizing the plasmonic nanoparticles present in HMGs^32^ and exploring their applications.^37^ UV-Vis spectroscopy has been reported as an effective tool in tuning the optical behavior of plasmonic NPs present within polymer microgels.^38^ Additionally, the catalytic behavior of nanoparticles and adsorption of microgels were investigated with UV-Vis spectroscopy, which allows for monitoring the progress of catalytic reactions and adsorption through spectrophotometry. For instance, Arif *et al.*^39^ utilized UV/Vis spectroscopy to characterize bimetallic (silver/cobalt)–poly(*N*-isopropylmethacrylamide) [Ag/Co–P(NIPMAM)] hybrid microgels. The formation of bimetallic (Ag/Co) nanoparticles within the P(NIPMAM) system was confirmed with the surface plasmon resonance (SPR) peak of silver nanoparticles at 416 nm. They examined the bimetallic nanoparticle stability in the P(NIPMAM), and investigated the tuning of their optical properties using UV-Vis spectroscopy. The same research group also explored the catalytic activity of this system for the catalytic reduction of methyl orange (MOr) in an aqueous medium using UV-Vis spectroscopy. Additionally, Pongsanon *et al.*^12^ employed UV-Vis spectroscopy to investigate the growth of gold (Au) nanoparticles with and without poly(*N*-isopropylacrylamide) P(NIPAM) microgels. They used different contents of Au nanoparticles during the formation of the Au–P(NIPAM) hybrid, and characterized these mixtures with UV-Vis spectroscopy. They also monitored the reduction of 4-nitrophenol (4NPh) with this system *via* UV-Vis spectroscopy. Furthermore, Rahman *et al.*^40^ investigated the adsorption capacity of poly(methacrylic acid) P(MAA) for methylene blue (MBl) and malachite green (MGr) from water. They investigated the adsorption content of dyes *via* UV-Vis spectroscopy with a decline in the *λ*_max_ value absorbance of dyes. Different adsorption factors such as the substance content and microgels were also studied, and the adsorption process was monitored *via* UV-Vis technique. Similarly, Wi *et al.*^41^ synthesized crosslinked microgels, and used them for the removal of Pd^2+^, Au^3+^, and Cr^6+^ ions through adsorption. They observed that physically crosslinked systems show superior adsorption capacity compared to others because of the presence of free –NH_2_ groups in the structures. These groups strongly interact with the metal cations and yield high adsorption from the medium. This adsorption was monitored *via* UV-Vis spectrophotometry with a decline in the absorbance peak. The shifting in the *λ*_SPR_ values of the hybrid microgels under various stimuli conditions could also be monitored with UV-Vis spectroscopy. This shifting indicates the swelling and deswelling behavior of microgels, which affects the refractive index value. Therefore, the *λ*_SPR_ values of the hybrids change from its normal position. A similar shifting behavior was reported by Arif^5^ on bimetallic (Ag/Ni)-containing hybrid systems in various pH and temperature values. Red shifting was observed upon increasing the temperature of the medium, owing to the deswelling behavior, resulting in increases in the refractive index. Meanwhile, blue shifting occurred under the opposite condition. Similarly, the red shifting was obtained with increasing pH, owing to increasing the diffusion and coagulation rates of the metal nanoparticles in the swelling state. This study was also evaluated by UV-Vis spectroscopy. The massive use of UV-Vis spectroscopy in the applications (adsorption and catalysis) and characterizations of both MGs and HMGs prompted us to review its functionality in research studies over the past few years.

This review extensively discusses the uses of UV-Vis spectroscopy in adsorption by microgels and hybrid microgels, along with kinetic parameters, the characterization of metal nanoparticles in microgels, the growth of metal nanoparticles within microgels, swelling and deswelling of hybrid microgels, shape identification of NPs in microgels, nanoparticle stability in microgels, and the optical behavior of HMGs. It also examines the behaviors of HMGs resulting from the presence of both organic and inorganic components in the HMGs. Furthermore, this review describes how UV-Vis spectroscopy is used to monitor catalytic reactions that occur in HMGs dispersion and determine different kinetic factors of the reactions. It concludes with a summary and future perspectives for more progress in this field.

## 2. Adsorption

Adsorption of different pollutants with MGs and HMGs was monitored with different instruments,^42–47^ from which UV-Vis spectroscopy is one of them.

### 2.1. Adsorption controlling factors

**Fig. 1:**
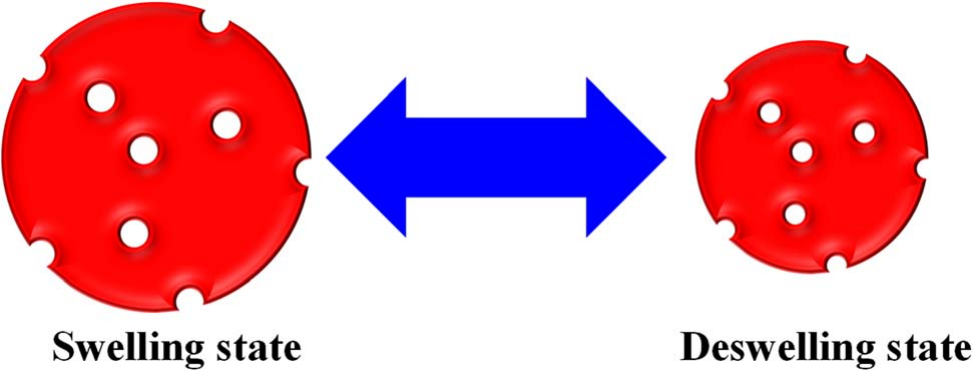
Swelling/deswelling behavior of microgels and hybrid microgels.

The monitoring of pollutant adsorption with microgel particles was estimated with the decline in the *λ*_max_ peak measured *via* UV-visible spectrophotometer with the passage of time. In this way, the absorbance peak of the pollutant appears in the UV-visible region. Initially, some content of pollutant is adsorbed onto or into the network of MGs or HMGs, and then separates the pollutant-loaded MGs or HMGs from the dispersion. The pollutant content then decreases in the medium, resulting in a decline in the *λ*_max_ peak. This peak decline was used to calculate the percentage removal of the pollutant with MGs or HMGs from the medium. For instance, Naseem *et al.*^48^ used poly(*N*-isopropylmethacrylamide–acrylic acid) P(NIPAM–AA) MGs for the adsorption of rhodamine-B (RDB), methylene blue (MBl), and Congo red (CRe) dyes. Greater contents of MBl and RDB were adsorbed with MGs than CRe at high pH and low temperature. The reason behind this phenomenon is that both MBl and RAB dyes are positively charged, while CRe is negatively charged. Under similar conditions, the structure of MGs is also negatively charged. Therefore, MGs have high affinity towards MBl and RDB because of the opposite charges and little affinity towards CRe (owing to the same charges). In this condition, the structure of MGs is hydrophilic in nature and present in the swelling state, owing to the same charges on their structure. The structural size of MBl is smaller than that of RDB. Therefore, a greater content of MBl was loaded than RDB, owing to the crosslinked network region. However, if the condition for adsorption is having a low pH and high temperature, then the structure of MGs is hydrophobic in nature. In this situation, more CRe content was removed by MGs than by MBl and RDB. They also examined the impact of the medium pH, temperature, dosage of MGs, dye content, and shaking time, as well as the effects of the pH and temperature of the medium on the deswelling/swelling behavior. More space is available in the swelling state, as shown in Fig. 1. Therefore, the adsorption capacity of both MGs and HMGs is higher in the swelling state than in the deswelling state. For example, Jabeen *et al.*^49^ examined the adsorptive capacity of acidic microgels. They reported on the adsorptive capacity of microgels against RB at various pH values. At high pH, the structure of MGs is present in the swelling state. In this condition, a greater content of RB is adsorbed by MGs, owing to the large available space and interactions; while a lesser content of RB is adsorbed at low pH, owing to the deswelling state.

**Fig. 2:**
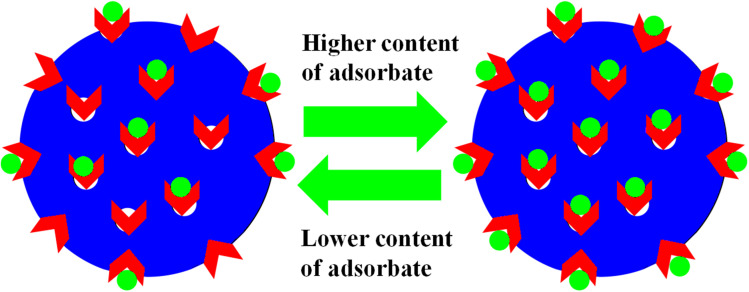
Adsorbate content impact on the adsorption capacity of both microgels and hybrids (red arrows stand for the functional groups and green dots correspond to pollutants).

Similarly, Rahman *et al.*^40^ studied the adsorptive potential of poly(methacrylic acid) P(MAA) MGs against cationic pollutants (malachite green (MGr) and MBl). They also investigated the content effects of dyes and P(MAA) on the adsorption capacity. The adsorption capacity increased with increasing P(MAA) content, owing to the increase in the availability of surfaces for the dye. On increasing the content of dye, the percentage removal initially increases and then becomes constant after attaining the equilibrium state. The dye molecules rapidly adsorbed on the P(MAA) surface with increasing concentrations because of decreasing the distance between the pollutant and MGs surface. No further adsorption occurs after attaining equilibrium, as shown in Fig. 2. The maximum percentage removal capacities obtained in MGr and MBl were 351 mg g^−1^ and 65 mg g^−1^, respectively. The greater percentage removal of MGr is due to the smaller surface area, which facilitates its penetration into the network of MGs. Meanwhile, the surface area of MBl is greater than that of MGr, resulting in the resistance in the diffusion rate of MBl molecules across the crosslinked structure. Therefore, compared to MBl, a greater content of MGr is removed from the medium with MGs.

**Fig. 3:**
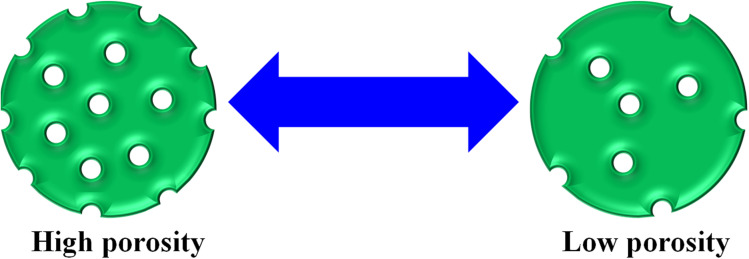
Pictorial representation of the porosity of microgels.

In this way, the removal of pollutants further increases with increasing porosity of the MGs surface. At high porosity, the pollutants can easily penetrate the network of MGs. A greater porosity means more empty space availability for adsorption, as shown in Fig. 3. Avais *et al.*^50^ reported the removal of MOr by porous MGs. They observed that a greater content of MOr has been removed with the presence of more porous MGs.

**Fig. 4:**
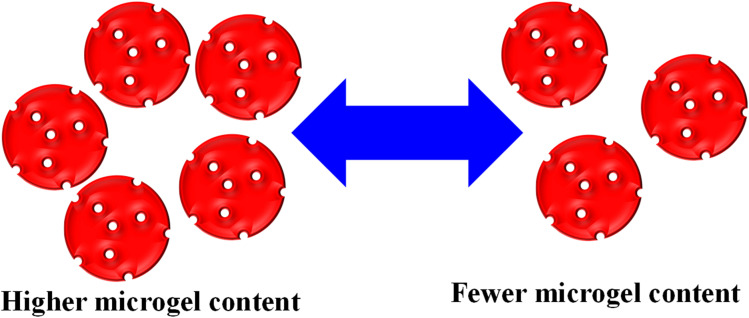
Availability of the empty space in microgels depends on the microgel content.

George *et al.*^51^ synthesized chitosan-based hybrid microgels, and used them for the adsorption of different metal cations. This hybrid system is highly sensitive towards H_2_O_2_, owing to the oxidizing ability of H_2_O_2_. The adsorbent content is also influenced by varying their content during the adsorption process. The availability of active sites increased with increasing content, as shown in Fig. 4. Rahman *et al.*^40^ used P(MAA) microgels for an adsorption study against MGr and MBl. The adsorption capacity of P(MAA) was increased with increasing P(MAA) content during the adsorption process. This increasing trend was attributed to the increasing empty space, which was available for dye adsorption.

Eqn (1) can be used to find the percentage removal of pollutants with MGs or HMGs.1
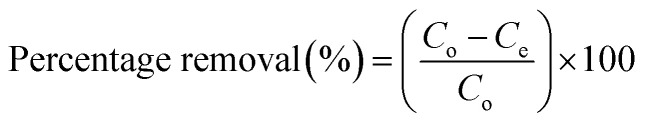
Here, *C*_e_ and *C*_o_ represent the equilibrium and initial content of pollutants in the medium, respectively. Furthermore, eqn (2) can be used to calculate the adsorption capacity (*q*_*t*_).2
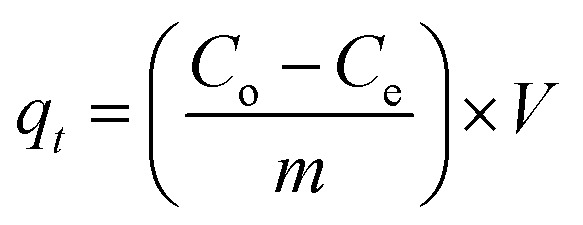
Here, ‘*m*’ stands for the mass of MGs or HMGs, and ‘*V*’ denotes the medium volume (in liters). Ribeiro *et al.*^52^ studied the adsorption of microgels for MBl. They noted the effect of temperature on the adsorption of MBl with MGs. The hydrodynamic diameter of MGs decreases upon the loading of MBl, as noted at the temperatures of 293 and 308 K. The functional groups of MGs interact with MBl, and no sites are available for interaction with water molecules. Therefore, the structure of MGs shifts from the swelling to deswelling state.

### 2.2. Adsorption isotherms

The microgels are added into the solutions of the pollutants. The pollutants moved towards the microgels from the bulk region and are adsorbed. After the adsorption of the pollutants, the microgels are removed from the medium, along with the adsorbed pollutants from the medium. The remaining solution was monitored with UV-Vis spectroscopy. The adsorption data are then evaluated by applying different adsorption isotherms, such as the Dubinin–Radushkevich (DRa), Freundlich (FLi), Langmuir (LMu), and Temkin (TKi) adsorption isotherms. The linear forms of DRa, FLi, LMu and TKi are defined as eqn (3)–(6), respectively.

3 ln *q*_e_ = ln *q*_DRa_ − *βε*^2^

4
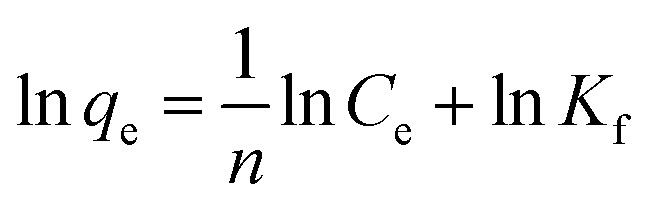
5
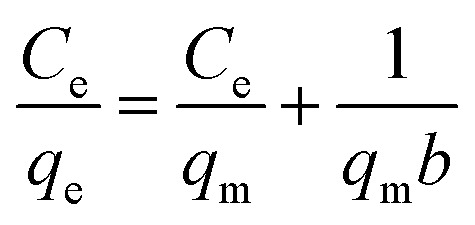

6 *q*_e_ = *K*_T_ ln *C*_e_ + *B*_T_

in these equations, *q*_e_*q*_DRa_, *C*_e_, and *q*_m_ stand for the pollutant content adsorbed on the MGs in equilibrium, theoretical saturation potential, pollutant content in equilibrium, and maximum capacity of adsorption by MGs, respectively, while *β*, *K*_f_, *b*, *K*_T_ and *B*_T_ stand for the constants of DRa, FLi, LMu, and TKi, respectively. These values are calculated from the slope and intercept values obtained from plotting the graph of these linear equations. The *R*^2^ values of these linear equations indicate the suitability of the isotherms on the adsorption data. Its value lies from 0 to 1. The most suitable isotherm is that which has the highest value among them. Pany *et al.*^53^ investigated the adsorption and releasing capacity of P(NIPAM–AA) MGs against MBl dye, monitoring the process *via* UV-Vis spectrophotometer. They also evaluated the adsorption data by applying different adsorption isotherms. The swelling and deswelling behavior of MGs was controlled with pH and temperature conditions, which also affected the interactions present between the MGs and pollutants, along with this swelling/deswelling behavior. The hydrophilic and hydrophobic interactions control the content adsorption of the pollutants. They found that 80% dye was adsorbed by MGs at 20 °C and pH = 7 in the swelling state, while the releasing content decreased from 87% to 63% in the deswelling state (at 50 °C and pH = 3) after 4 cycles. Similarly, Kubiak *et al.*^54^ investigated the adsorption capacity of MGs against several derivatives of bisphenols. The adsorption capacity of MGs was obtained in the 55–160 mg g^−1^ range for these bisphenols. The adsorption data follow the LMu isotherms.

### 2.3. Adsorption kinetics

Similarly, the kinetics of adsorption was also evaluated by applying eqn (7)–(10). These equations are linear forms of the pseudo-1^st^ order (P1Or), pseudo-2^nd^ order (P2Or), Elovich (EVi), and intra-particle diffusion (IPDi) models, respectively.

7 ln(*q*_e_ − *q*_*t*_) = −*k*_1_*t* + ln *q*_e_

8
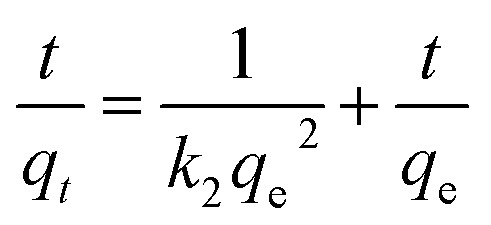
9
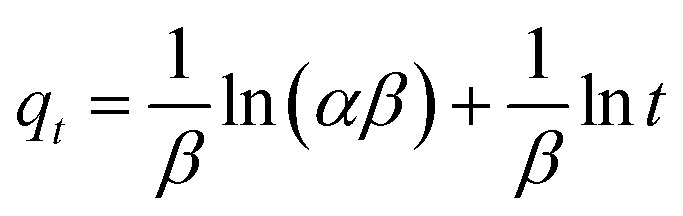

10 *q*_*t*_ = *k*_ipd_*t*^1/2^ + *C*

Here, *q*_e_, *q*_*t*_, and *t* stand for the content of pollutant adsorbed per-unit-mass of MGs at equilibrium, at specific time *t*, and time of adsorption, while *k*_1_, *k*_2_, and *C* are the constant values of the P1Or, P2Or and IPDi models, respectively. These values are also achieved from the intercept and slope values after plotting the graphs by these equations.

For example, Atta *et al.*^55^ investigated the adsorption capacity of poly(2-acrylamido-2-methylpropane sulfonic acid–acrylic acid) P(AAMPSA–AA) MGs, Fe_3_O_4_–P(AAMPSA–AA), and Ag–P(AAMPSA–AA) HMGs against MBl. The removal of MBl was studied with UV-Vis spectrophotometry. They also investigated the kinetics of the adsorption, along with tuning factors, like the contact time, pH, content of pollutants, and MGs. They found that the adsorption rate of MBl was in the following order:P(AAMPSA–AA) < Fe_3_O_4_–P(AAMPSA–AA) < Ag–P(AAMPSA–AA)

The reason behind this is that the size of the Ag nanoparticles is smaller than that of Fe_3_O_4_. Therefore, the surface area is greater, and more available sites are present for the interaction of the pollutants. The physicochemical interaction supports these adsorption trends.

Similarly, Kubiak *et al.*^54^ investigated the kinetics of adsorption of bisphenols on MGs. According to their study, the P2Or model was the best fit on the adsorption mechanism. The selectivity on the adsorption of the pollutants can be done on the basis of the charged structure of MGs. Under these conditions, the pH of the medium plays an important role in creating charge in the network of MGs. In this way, attraction takes place where opposite charges are present on the MG networks and pollutant, while repulsion occurs where the same charges are present on both. Similar selective adsorption trends were reported by Atta *et al.*,^56^ Chen *et al.*,^57^ and He *et al.*^58^

The main point for the adsorption study by microgels is because the absorbance value of pollutants lies in the ultraviolet, visible, and near infra-red regions. The pollutants that do not have absorbance values in these regions cannot be monitored with UV-Vis spectrophotometry. The visible region is the best for adsorption monitoring. The adsorption parameters and kinetics of the pollutants are studied using the decline in the *λ*_max_ value. Therefore, the adsorption of the pollutants should be present in the visible region.

## 3. Optical behavior

Optical behavior of HMGs can be possible if the HMGs contain Au or Ag nanoparticles in MGs. The MGs do not show optical behavior. Therefore, it is applicable to only HMGs. This optical property also helps in the formation, shape, stability, size, and growth of NPs under different environments.

### 3.1. Identification of nanoparticles in microgels with UV-Vis spectrophotometer

It is widely understood that plasmonic NPs absorb radiation within the visible and near-infrared (NIR) regions. This property depends on their shape and size. This absorption is attributed to the collective surface electron oscillation of the nanoparticles, which is called surface plasmon resonance (SPR). The dispersion of plasmonic nanoparticles results in one or more peaks, which can provide valuable evidence regarding the size distribution, shape, and size of the NPs. This is the reason “why UV-Vis spectroscopy is commonly used to characterize plasmonic NPs present in smart MGs”.

**Fig. 5:**
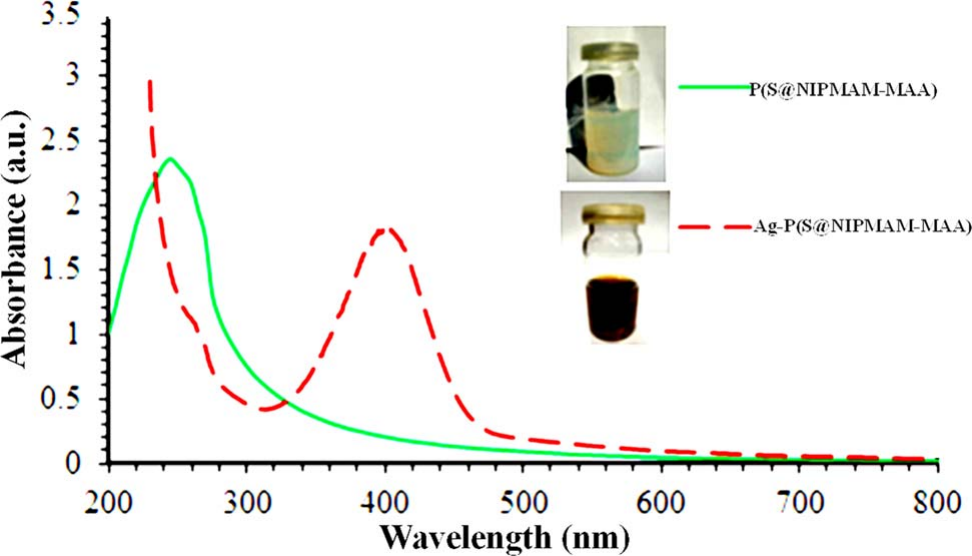
UV-Vis spectrum of both P(S@NIPMAM–MAA) and Ag–P(S@NIPMAM–MAA) (reproduced from ref. 61 with permission from Elsevier, copyright 2021).^61^

For instance, Zahid *et al.*^59^ utilized UV-Vis spectroscopy to characterize P(NIPAM–AAMPSA) MGs and Ag–P(NIPAM–AAMPSA) HMGs. They performed UV-Vis spectroscopy on diluted dispersions of pure P(NIPAM–AAMPSA) microgels and Ag–P(NIPAM–AAMPSA) hybrids microgels in the 200–800 nm wavelength range. No peak was observed from the P(NIPAM–AAMPSA) dispersion, while the Ag–P(NIPAM–AAMPSA) dispersions showed a single peak with a narrow range at 401 nm. The presence of this single peak at a specific wavelength (401 nm), called the surface plasmon resonance (SPR) wavelength (*λ*_SPR_), indicated the successful loading of spherical nanoparticles into the structure of MGs. The small width of this peak indicated a narrow size distribution of the NPs. Khan and his group^60^ have reported on the UV-Vis analysis of silver nanoparticles (Ag NPs) in poly(*N*-isopropylacrylamide-2-hydroxyethyl acrylate) [P(NIPAM–HEAc)] HMGs to assess their optical nature. HEAc was added to increase the loading of Ag^+^ ions into the sieves. The –OH groups of HEAc generate strong interaction between Ag^+^ ions and the MGs network. Therefore, the content of Ag NPs in MGs was controlled with HEAc. UV-Vis measurements were used to determine the size of NPs. The single peak of HMGs indicated that Ag NPs are spherical in shape with a narrow distribution size. Similarly, Hussain *et al.*^61^ investigated the synthesis of Ag NPs in P(S@NIPMAM–MAA) core–shell MGs. They observed that no peak was present in the microgels, while a peak was present in Ag–P(S@NIPMAM–MAA) HMGs, as shown in Fig. 5.

**Fig. 6:**
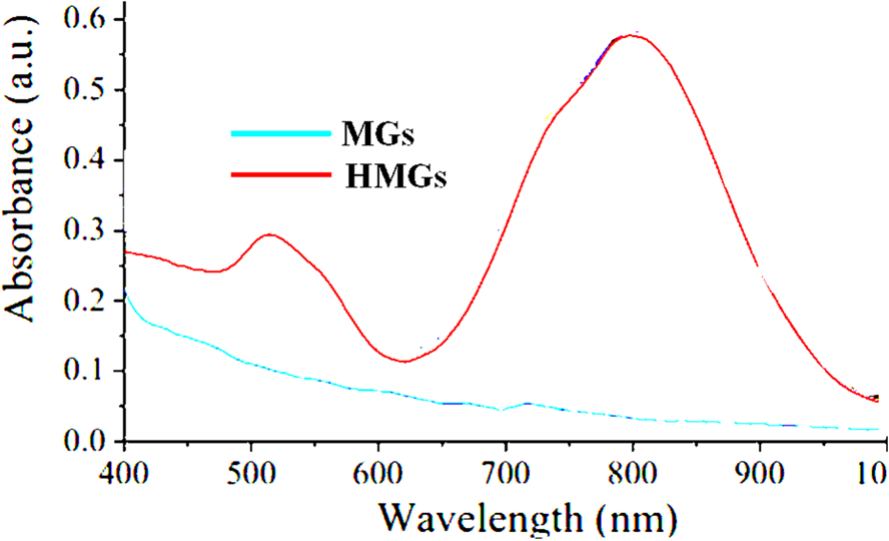
UV-Vis spectra of Au nanorod-containing microgels and pure MGs (reproduced from ref. 62 with permission from Elsevier, copyright 2019).^62^

If two peaks of *λ*_SPR_ of HMGs are obtained, this indicated that the shape of the particles is rod-like. From these two peaks, the one appearing at shorter wavelengths is known as the transverse-surface-plasmonic-resonance (TSPR), while the other at the longer wavelength is called the longitudinal-surface-plasmon-resonance (LSPR). Wang *et al.*^62^ synthesized Au nanorods containing hybrid microgels. Because the Au particles are present in the nanorod-like shape in the microgels, two peaks were observed in the HMGs spectrum, while no peak appeared in MGs, as shown in Fig. 6.

Khan and his coworkers^63^ analyzed Au particles containing HMGs *via* UV-Vis spectroscopy. The HMGs dispersion showed two peaks at 514 nm and 689 nm for TSPR and LSPR, respectively, in the UV-Vis spectrum. The presence of bimetallic (these (Au, Ag) nanoparticles along with other metal) NPs was also characterized by UV-Vis analysis. The HMGs still show a peak in the UV-Vis spectrum. However, the intensity of the plasmonic peaks depends on the content of Au and Ag nanoparticles in MGs. Raza *et al.*^64^ analyzed the bimetallic (Ti/Ag) NPs containing HMGs. A clear peak of the Ag nanoparticles was obtained in the HMGs spectrum. The lower intensity of the peak was because of the Ti particles, which caused a decrease in some intensity. The longer wavelength shifting in the UV-Vis spectrum indicates that the size of the NPs are larger than that of NPs with shorter wavelengths. In this way, the NPs size can also be determined with UV-Vis spectroscopy. Arif *et al.*^33^ also investigated the UV-Vis spectra of HMGs and MGs. Only one peak was obtained at 520 nm with a narrow width range. This indicates that the Au NPs are present in spherical form with a narrow size range. Chen *et al.*^65^ synthesized modified surface-Au nanorods around the microgels, and monitored the stability of the Au nanorods in various temperatures and pH values. The hybrid system showed two peaks to indicate the rod-like shape of the Au particles *via* UV-Vis spectroscopy. They also observed that the intensity of *λ*_SPR_ of the Au nanorods increased with increasing temperature. This only decreases the inter-particle distance between the Au nanorods. They also noted that the *λ*_SPR_ value changes with the changing content of monomers, owing to variations in the refractive index. The hybrid microgels have excellent stability in different solvents, which indicate that the hybrid systems can be used for a long time.

### 3.2. NPs stability in microgels

**Fig. 7:**
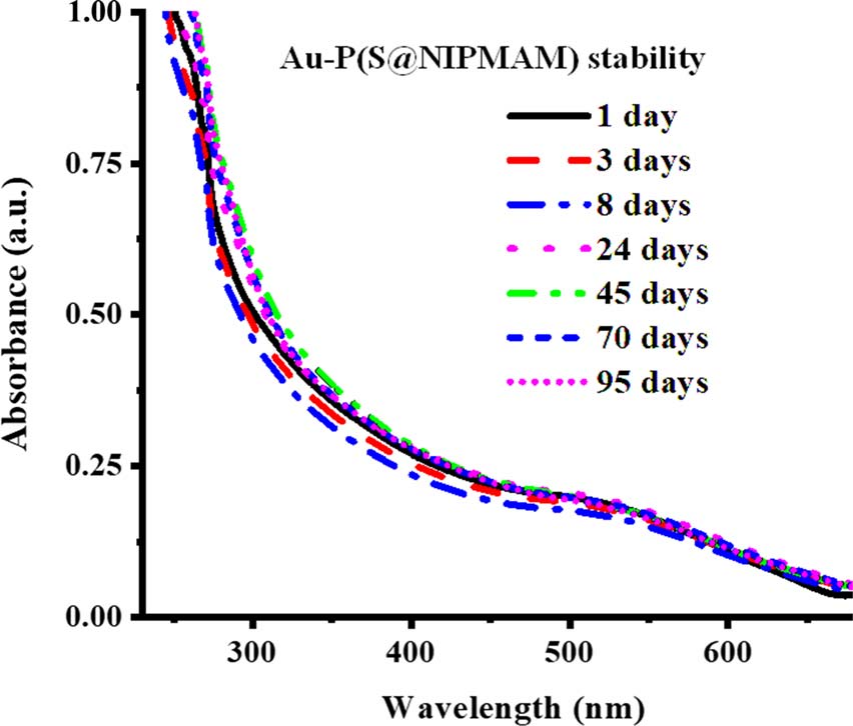
Au NPs stability in P(S@NIPMAM) microgels (reproduced from ref. 66 with permission from Elsevier, copyright 2022).^66^

Metal nanoparticles incorporated into the network of MGs have been observed to exhibit long-term stability because of the donor–acceptor interactions of metal nanoparticles with the functional groups of microgels, as described in the literature. This long-term stability of NPs within the network of microgels is verified with UV-Vis spectroscopy. The stability investigation involves monitoring the surface plasmon resonance (SPR) wavelength of the hybrid microgels with respect to the storage time. To achieve this, a diluted dispersion of HMGs is kept in the dark under ambient temperature, and their UV-Vis spectra are periodically scanned to note any shift in the *λ*_SPR_ value. The absence of a shift in the *λ*_SPR_ value over time signifies the stability of the metal nanoparticles within the structure of MGs. For instance, Arif *et al.*^66^ investigated the stability of Au nanoparticles in the poly(styrene@*N*-isopropylmethacrylamide) P(S@NIPMAM) core–shell MGs with UV-Vis technique, as shown in Fig. 7. To perform this study, a diluted dispersion of Au–P(S@NIPMAM) HMGs was kept in the vial, which is covered with aluminum foil. The UV-Vis spectra of the Au–P(S@NIPMAM) HMGs dispersion were periodically scanned for up to 95 days after HMGs synthesis. They did not observe any shifting in the value of *λ*_SPR_ at 520 nm, confirming the stability of the Au nanoparticles for long-term practical applications. Farooqi *et al.*^67^ synthesized Ag NPs in homogeneous MGs, and examined the Ag NPs stability in the structure of MGs. They observed the spectra of HMGs obtained by UV-Vis spectroscopy for up to 42 days. They also reported that no shifting was observed in the *λ*_SPR_ value, resulting in the long-time stability of HMGs.

This scanning method of HMGs, which is applied to check the stability of NPs in MGs, is only useful for Au and Ag NPs because of their active surface-plasmon-resonance behavior. The stability of other hybrid systems, which do not exhibit active surface-plasmon-resonance behavior, cannot be examined using this procedure. The stability of those hybrid microgels is typically investigated by applying the freshly prepared and stored hybrid microgels against a particular application like catalytic reduction. In this case, the reduction of the same pollutant is studied with freshly synthesized HMGs and stored. If the same catalytic behavior of HMGs is obtained from the stored HMGs as well as fresh, then the nanoparticles are stable in the structure. Meanwhile, if the catalytic behavior of HMGs is not present, this indicates that the HMGs are not stable for a long time. The catalytic behavior of HMGs can be also studied with UV-Vis spectroscopy. For example, Arif *et al.*^68^ investigated the stability of Pd NPs in core–shell MGs in a similar way. They studied the stability of the catalytic activity of Pd NPs in core–shell MGs against 4-nitroaniline (4NAn). The hybrid system showed almost the same catalytic behavior after three months, as that shown by the freshly prepared HMGs. The catalytic behavior was also investigated *via* UV-Vis spectroscopy. The results showed that the HMG system was stable in MGs. In this approach, the stability of all types of hybrids can be investigated with UV-Vis spectroscopy because all types of hybrid microgels are catalytically active. George *et al.*^51^ studied the stability of Ag nanoparticles in chitosan-based microgels. They examined the stability of NPs and hybrids under acidic and basic conditions. The hybrid microgels showed excellent stability under both media conditions.

### 3.3. Smart behavior

The microgels that show swelling/deswelling behavior under stimuli conditions are called smart microgels, and the swelling/deswelling behavior is called smart behavior. This swelling/deswelling behavior occurs due to the hydrophilic and hydrophobic nature. In the hydrophilic nature, the microgels are present in the swelling state because the network has greater interactions with water molecules as compared to their functional network. Meanwhile, stronger interactions are present between the functional networks themselves as compared to water molecules in the hydrophobic interactions. Hence, the water molecules come out from the network and the microgels shift to the deswelling state. This swelling/deswelling behavior changes the crosslinking density of microgels around nanoparticles; hence, there is an effect on the *λ*_SPR_ value of the hybrid microgels. This smart behavior of the hybrid microgels controls the refractive index, which responds to the change in the *λ*_SPR_ value. This shifting can be monitored with UV-Vis spectroscopy.^69^

**Fig. 8:**
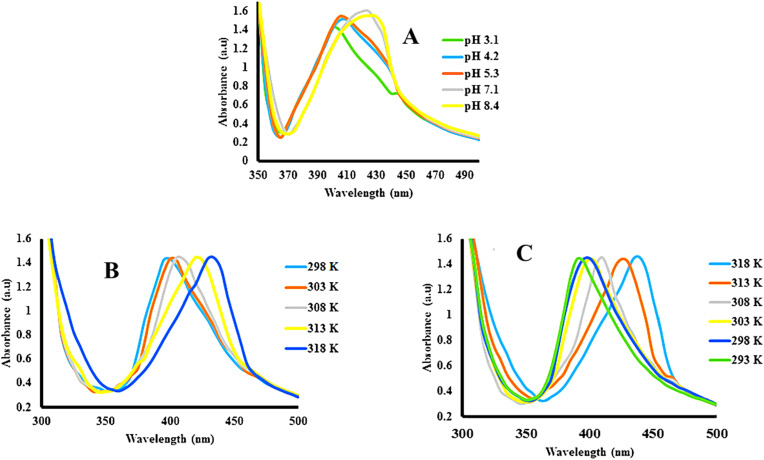
*λ*_SPR_ value shifting of Ag–P(S@NIPMAM–MAA) with (A) increasing pH, (B) increasing temperature, and (C) decreasing temperature (reproduced from ref. 69 with permission from Wiley, copyright 2021).^69^

The shifting in the *λ*_SPR_ value mostly changes with changing pH (Fig. 8(A)) and temperature (Fig. 8(B) and (C)) of the medium. The interaction between the structure of the hybrid microgels and water depends on the kinetic energy of the molecules. As the kinetic energy of the molecules increases, the strength of interactions between the two molecules decreases. Therefore, the structure of the hybrid microgels shifts from hydrophilic to hydrophobic in nature, and water molecules come out from the network. Hybrid microgels convert from the swelling to deswelling state. For example, Hussain *et al.*^69^ studied the temperature effect on the swelling/deswelling behavior of Ag–P(S@NIPMAM–MAA) HMGs. They changed the temperature within the range of 293–318 K, and observed the shifting in the *λ*_SPR_ value, as shown in Fig. 8(B) and (C). The *λ*_SPR_ value was altered in the range of 400–435 nm. The *λ*_SPR_ peak value appeared at 400 nm at 293 K, owing to the swelling behavior. On increasing the temperature, the inter-NPs distance decreased gradually, along with increasing refractive index, owing to the deswelling nature of NIPMAM. On decreasing in temperature, the reverse behavior of the *λ*_SPR_ value was noted. This phenomenon indicates that the coagulation of NPs does not occur during the increasing of temperature. The shifting occurred because of the inter-NPs distance and refractive index value. They also studied the pH effect on the swelling/deswelling behavior, as shown in Fig. 8(A). Similar increasing trends in the *λ*_SPR_ value of the bimetallic hybrid system was reported by one of our groups.^5^ He also reported that the *λ*_SPR_ value of this system increased because of the deswelling nature. Deswelling of hybrid microgels increases the refractive index of hybrid microgels, which then causes the shifting of the *λ*_SPR_ value.

Similarly, the hybrid microgels exhibited changes in the *λ*_SPR_ value with varying pH values of the medium. The hybrid microgels are divided into two categories: (1) acidic hybrid microgels and (2) basic hybrid microgels. Acidic hybrid microgels are those which have the ability to donate the protons from their structure, while basic hybrid systems are those that have the ability to accept protons. The structure of the acid hybrid microgels shifts from neutral to anionic (deprotonated) form (pH ≥ p*K*_a_) after releasing the protons from their structure. Therefore, repulsive forces take part in the structure of hybrid systems owing to the same charges, and the network converts from deswelling to swelling state on increasing the pH of the medium (pH ≥ p*K*_a_). On the other hand, the structure of the basic hybrid systems accepts the protons at pH ≤ p*K*_a_ value, and shifts into the cationic (protonated) form. In this form, repulsive forces take place between the structure of the protonated hybrid microgels owing to the similar charges. Therefore, the network of basic hybrid systems shifts from the deswelling to swelling state at pH ≤ p*K*_a_.

Siddiq *et al.*^70^ investigated the pH changing impact on the swelling/deswelling behavior of Ag NP-based acid hybrid microgels. They monitored the hybrid microgel dispersion at different pH (2, 6, 10) values *via* UV-Vis spectrophotometer. They found that the *λ*_SPR_ value had shifted from 394 to 403 nm with increasing pH from 2 to 10. This increasing in the *λ*_SPR_ value indicated that the network of hybrid microgels converts from deswelling to swelling state with increasing medium pH value. Because the structure of the hybrid system released the protons from their structure by increasing the pH of the medium, this resulted in electrostatic repulsion among the network (owing to the same negative charged parts). During this swelling, some nanoparticles diffuse across the network and coagulate. Therefore, the *λ*_SPR_ value increased during increasing the pH of the medium in acidic hybrid microgels. Similar increasing trends of the *λ*_SPR_ value of acidic hybrid microgels have been reported by Arif,^5^ and Hussain *et al.*^69^

### 3.4. NPs content in hybrid microgels

The *λ*_SPR_ value of hybrid microgels also shifts with the content of metal nanoparticles in microgels. If the content of nanoparticles is higher, then a red shift and higher intensity peaks are obtained with the higher *λ*_SPR_ value, owing to coagulations. When the content is higher, then the inter-particle distance decreases and coagulation occurs. Therefore, red shifting occurs. If the content of NPs is small, then small size particles are formed. The distance between these particles is large. Therefore, coagulation will not occur, and blue shifting occurs in this condition. For example, Li *et al.*^71^ studied the optical study of mono- and bi-metallic nanoparticles containing hybrid systems. They reported that the *λ*_SPR_ value of Ag nanoparticles increased after the formation of microgels around these nanoparticles, owing to the increasing refractive index. They also reported that the intensity of the peak increases with increasing NP content, along with red shifting, owing to the NPs coagulation and decreasing the distance between NPs. The medium also affects the shifting of *λ*_SPR_ value, as the solvent will either have affinity towards the network of microgels or not. If the solvent has a high refractive index value, then the *λ*_SPR_ value of the hybrid microgels will show a red shifting and *vice versa*. They ran the dispersion in CS_2_, tetrahydrofuran (THF), acetone, and water. The refractive index values of these solvents are 1.63, 1.42, 1.36, and 1.33, respectively, while the *λ*_SPR_ value of the hybrid microgels in these solvents were noted at 467 nm, 460 nm, 454 nm, and 446 nm, respectively. This shifting was obtained because of the refractive index of the solvent, and the deswelling of the hybrid microgels in these solvents. Maximum deswelling occurs in CS_2_, owing to the strong interactions between their structure of hybrid microgels. Meanwhile, a high interaction is present with the water molecules and the structure of the hybrid microgels. Therefore, hybrid microgels are present in swelling state in this condition. Melinte *et al.*^72^ also reported on a similar shifting in the *λ*_SPR_ value with increasing content of Ag or Au in the hybrid microgels. Pany *et al.*^73^ studied the integration of Ag nanoparticles into microgels. Initially, the particle size in the microgels was small. Therefore, the *λ*_SPR_ peak was shorter with a small intensity. This intensity increased with the passage of time owing to coagulation with longer wavelengths.

The optical study of hybrid microgels and microgels is very helpful to identify the presence of nanoparticles with UV-Vis spectroscopy. The optically active metal nanoparticles like Au and Ag NPs show a characteristic peak, while this peak is absent in the microgels. This peak from the nanoparticles is further used for identification of the stability, temperature effect, pH effect, and solvent effect on the hybrid microgels, and all these studies can be achieved *via* UV-Vis spectroscopy.

## 4. Catalytic investigation of reactions by hybrid microgels

All types of hybrids show catalytic activity because of the existence of metal nanoparticles in the microgels. These reactions are mostly monitored by UV-Vis spectroscopy.^67,74–77^

### 4.1. Reaction monitoring

UV-Vis spectroscopy is a powerful tool for investigating the catalytic performance of HMGs in model reactions. The 4-nitrophenol (4NPh) reduction is commonly employed as a catalytic model reaction to assess the catalytic efficiency of nanoparticles incorporated in the network of microgels.^38,78–80^ Additionally, reduction of other nitroarenes, such as 4-nitroaniline (4NAn),^68^ 3-nitroaniline (3NAn),^81^ 2-nitroaniline (2NAn),^82^ 2-nitrophenol (2NPh),^75^ 3-nitrophenol (3NPh),^64^ nitrobenzene (NBe),^83^ 2-chloronitrobenzene (2CNBs),^68^ and 4-chloronitrobenzene (4CNBe),^33^ along with dyes, such as eosin-Y (EY),^84^ MOr,^29^ CRe,^85^ rhodamine-B (RB),^86^ MBl,^87^ Eriochrome black T (EBT),^88^ methyl red (MRe),^5^ and brilliant (BLi)^89^ has been observed using UV-Vis spectroscopy.

The reduction progress for 4NPh and its corresponding 4NAn product is analyzed *via* UV-Vis spectrophotometry. The *λ*_max_ value of 4NPh appeared at 400 nm, while its value appeared at 300 nm for 4NAn. The 4NPh reduction was conducted along with hybrid microgels and sodium borohydride (reducing agent), and the spectra of the reaction spanned from 200 nm to 600 nm. As time progressed, a decline in the absorbance at 400 nm was achieved owing to the 4NPh reduction. At the same time, a new peak appeared at 300 nm owing to 4NAn formation. Many studies have explored the catalytic activity of various hybrid systems in the 4NPh reduction *via* UV-Vis spectrophotometry. For instance, Arif *et al.*^68^ utilized UV-Vis spectrophotometry to monitor the 4NAn reduction with a system of Pd nanoparticles incorporated into silica@NIPAM–MAA core–shell HMGs in an aqueous medium. Similarly, Shahid *et al.*^46^ examined the 4NPh reduction with NaBH_4_ (reducing agent) along with Co NP-based HMGs, also using UV-Vis spectroscopy. They noted that the peak of the 4NPh absorbance showed a new peak at 300 nm in the UV-Vis spectrum, which was obtained by the decline at 400 nm, indicating the 4NPh conversion to 4NAn. Arif *et al.*^26^ also used Cu NPs incorporated into poly(*N*-vinylcaprolactam–methacrylic acid) Cu–(P(VCLa–MAA) HMGs as a catalyst for the reduction of 4NPh, 4NAn, EY, RB, and Cr^6+^ ions. Cu NPs embedded in the microgel exhibited significantly higher catalytic efficiency for 4NPh reduction. Additionally, numerous researchers have reported on the monitoring of numerous catalytic reactions performed with HMGs using UV-Vis spectrophotometry.

### 4.2. Rate constant value

The observed rate constant (*k*_obs_) of the catalytic reduction reactions performed in the dispersion of HMGs along with NaBH_4_ has been extensively calculated with the help of UV-Vis spectroscopy, which is employed as detector for monitoring the reaction.^90^ During this calculation, the decline in the absorbance with time was used for the kinetic study. The reaction time (*T*_rea_) corresponds to the time window when the value of absorbance gradually decreases with time, and the induction time (*T*_ind_) corresponds to the time window in which the decline in the absorbance does not start. The induction time (*T*_ind_) is the period that is required for the reactant to reach the surface of the nanoparticles. The time in which no decline in the absorbance occurs after the reaction time is called the completion time (*T*_com_). For instance, recent studies, including our own,^68^ have reported the determination of *k*_obs_ for the 4NAn reduction with the temperature-sensitive HMGs. In this performance, the induction time, reaction time, and completion time were also noted, and the reaction time was employed for the *k*_obs_ value calculation. To determine the *k*_obs_ for this catalytic reaction, the natural logarithm of the ratio of the absorbance values (ln(*A*_*t*_/*A*_o_)) was used to draw a graph against the reaction time using the eqn (11) and (12).11
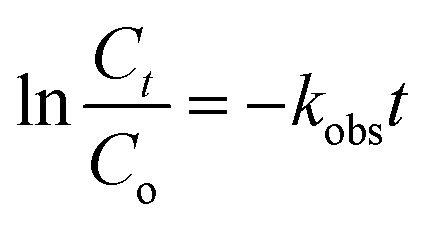
12
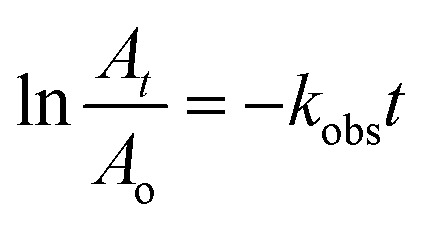
Here, *A*_*t*_ represents the 4NAn absorbance at any given time, while *A*_o_ denotes the absorbance value at initial time (zero time). Mustafa *et al.*^91^ have calculated the *k*_obs_ value for the 4NPh reduction with Ag–P(HEMA–NIPAM) HMGs catalysts through UV-Vis spectrophotometry. The *T*_ind_ decreased as the content of the catalyst increased. *T*_ind_ refers to the period during which the reaction did not commence despite the catalyst addition to the reaction. The 4NPh absorbance was noted at 400 nm under various time intervals throughout the reaction progress. The *k*_obs_ value was evaluated from the linear portion of the ln(*A*_*t*_/*A*_o_) *vs.* time graph, revealing that ln(*A*_*t*_/*A*_o_) decreased with increasing *T*_rea_. The *k*_obs_ value increased from 0.127 to 0.232 min^−1^ with increasing temperature from 294 K to 303 K, while this value decreased to 0.153 min^−1^ upon further increasing the temperature. This decreasing trend was obtained because of the shifting of the hybrid microgels from the swelling to deswelling state. Therefore, the diffusion rate decreases, which reduces the *k*_obs_ value. Similarly, Arif *et al.*^33^ have employed the Au NP-based core–shell HMGs for the reduction of 4NPh. They studied the 4NPh reduction under various values of temperature, HMGs content, 4NPh concentrations, and NaBH_4_ content. The value of *k*_obs_ increased with increasing content of HMGs or NaBH_4_, or temperature, or 4NPh to a particular level. However, after that value, a decline in *k*_obs_ was achieved for the NaBH_4_ and 4NPh content. They also reduced various other nitroarenes under similar circumstances of pressure, and the reactions were monitored with UV-Vis spectroscopy.

### 4.3. The *k*_obs_ value depends upon the following factors

The effects of various factors, such as the catalyst dose, reducing agent concentration, substrate type, temperature, pH, and NPs content in microgels, on the *k*_obs_ value have been investigated using UV-Vis spectroscopy. These factors control the diffusion rate or surface area of nanoparticles, which are the point for the *k*_obs_ value.

**Fig. 9:**
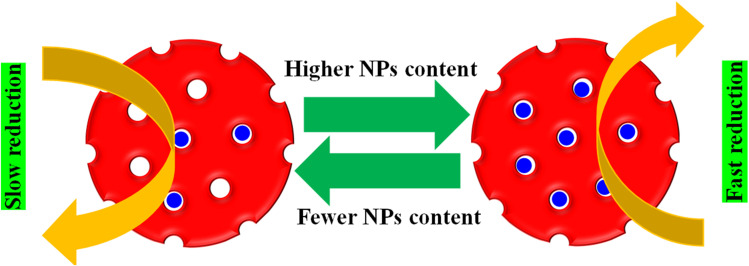
The *k*_obs_ value depending on the NPs content in microgels.

The NPs content in MGs also has an impact on the *k*_obs_ value for the reduction of pollutants. If the content of NPs in the MGs structure is higher, then the distance between the pollutants and NPs surface is smaller, resulting in less NPs containing HMGs, as shown in Fig. 9.

Therefore, the pollutants rapidly reached the NPs surface and were reduced rapidly. Gancheva *et al.*^92^ have synthesized porous and non-porous hybrid microgels. They investigated their catalytic performance against 4NPh. A greater content of NPs is present in the porous HMGs, while a lower amount is observed in the non-porous HMGs. Therefore, the porous HMGs showed better catalytic performance than the non-porous HMGs.

The content of HMGs also influences the *k*_obs_ value of the catalytic reactions. When the HMGs content is used in higher amounts, then the value of *k*_obs_ is greater, as given in Table 1. The reason is that at a higher contents of HMGs, the distance between the NPs surface and the reactant decreases. Therefore, the reactants rapidly reached the NPs surface and were reduced rapidly. In addition, the surface of NPs increased with increasing HMGs content. Therefore, there is a greater reactant content that is adsorbed on the NPs surface in a short time and rapidly reduced into the product. This reduction process can also be checked *via* UV-Vis spectroscopy. For example, Farooqi *et al.*^67^ have studied the catalytic behavior of A–P(NIPAM–AA) for MGr reduction at various MHGs contents. The performance was monitored *via* UV-Vis spectroscopy, and results showed that the *k*_obs_ value increased with increasing HMGs content because of the increase in the HMGs adsorption sites. Similar observations have been reported by various other researchers in the literature.^68,93,94^

**Table 1:** *T*_rea_, *T*, and *k*_obs_ values obtained for 4NAn reduction under different contents of 4NAn, HMGs, NaBH_4_, and temperature (reproduced from ref. 68 with permission from Elsevier, copyright 2024)^68^

Reaction conditions	*T* (K)	NaBH_4_ (mM)	Pd–P(Si@CS–NIPAM–MAA) (μg mL^−1^)	4NAn (mM)	*T*_rea_ (min)	*k*_obs_ (min^−1^)
NaBH_4_	302	1.21	2.29	0.056	15–21	0.92
	302	1.95	2.29	0.056	12–17	1.00
	302	2.67	2.29	0.056	8–13	1.17
	302	3.54	2.29	0.056	5–9	1.34
	302	4.29	2.29	0.056	3–8	1.16
	302	5.09	2.29	0.056	3–9	0.91
	302	5.95	2.29	0.056	1–7	0.80
Pd–P(Si@CS–NIPAM–MAA)	302	3.54	1.19	0.056	8–13	0.89
	302	3.54	2.29	0.056	4–10	0.95
	302	3.54	3.59	0.056	4–9	1.04
	302	3.54	4.79	0.056	3–8	1.12
	302	3.54	5.99	0.056	1–6	1.21
4NAn	302	3.54	2.29	0.038	2–6	0.97
	302	3.54	2.29	0.044	2–6	1.02
	302	3.54	2.29	0.050	2–6	1.10
	302	3.54	2.29	0.056	1–5	1.27
	302	3.54	2.29	0.062	3–7	1.16
	302	3.54	2.29	0.068	3–8	1.12
	302	3.54	2.29	0.074	3–8	1.06
Temp.	297	3.54	2.29	0.056	2–9	0.57
	301	3.54	2.29	0.056	2–8	0.72
	305	3.54	2.29	0.056	1–6	0.95
	309	3.54	2.29	0.056	1–6	0.87
	313	3.54	2.29	0.056	1–7	0.75

The *k*_obs_ value depends on the substrate content. Initially, the value increases with increasing substrate content up to a specific level, while it decreases after further increasing from the content of substrate as given in Table 1. Initially, the distance decreases with increasing substrate content. Therefore, the diffusion rate increases with the substrate content and is reduced rapidly. Meanwhile, the number of collisions of the reactants also increases with increasing substrate content. This number of collusions resists the approach of the reactants to the NPs surface. This resistance dominates the approach of substrate to NPs surface at a specific level of substrate content. Therefore, the reduction rate decreases after that content. This study is also observed *via* UV-Vis spectroscopy. For example, Ahmad *et al.*^95^ have studied the catalytic performance of Ag NPs in homogeneous HMGs. They reduced the 4NPh and monitored the progress in UV-Vis spectroscopy. Similar results were obtained for the kinetic observation. Hussain *et al.*^96^ have also reported similar results during the reduction of MBl.

The pH value of the medium also controls the *k*_obs_ value during catalytic reduction of substrate by HMGs. Basically, the pH value of the medium controls the swelling and deswelling behavior of both acidic and basic hybrid microgels, as discussed in Section 3.3. In this way, the pH controls the diffusion rate of the reactant to the NPs surface. The acidic hybrids convert from the deswelling to swelling state with increasing pH value, owing to deprotonation (negative charged structure formation), while basic hybrids converted the swelling state to deswelling on increasing the pH value owing to deprotonation (positive to neutral structure formation). Hence, the pH effect on the *k*_obs_ value and these catalytic reactions are monitored *via* UV-Vis spectroscopy. For instance, Rahman *et al.*^97^ investigated the catalytic performance of Ag–P(MAA) against 4NPh. They observed the pH effect on the *k*_obs_ value. Here, Ag–P(MAA) is acidic hybrid microgels. Therefore, this hybrid system donates their protons during the pH increase, and converted into neutral to anionic formed hybrid microgels. In this form, the hybrid microgels are in the swelling state. Therefore, the diffusion of the reactant was greater than the deswelling forms. So, the *k*_obs_ value increases with increasing pH.

The content of a reducing agent like NaBH_4_ also affects the *k*_obs_ value, as given in Table 1. The NaBH_4_ provides hybrid molecules that are used as reactants to reduce the substrate from medium. The surface of NPs is the place at which the substrate reduction occurs in the presence of both NabH_4_ and HMGs. The *k*_obs_ value increased at the start with increasing NaBH_4_ content to a particular content. On further increasing the content of NaBH_4_, the *k*_obs_ value decreases. Initially, the *k*_obs_ increases owing to the decreased distance between the reactants and NPs surface. Therefore, the reactants rapidly reached the surface of NPs. On further increasing, the surface of NPs is mostly covered with hydrogen molecules and there is no available space for adsorption of substrate. Therefore, the *k*_obs_ decrease on increasing after a particular content of NaBH_4_. This study was also checked with the help of UV-Vis spectroscopy. For instance, Arif *et al.*^39^ employed bimetallic NPs decorated homogeneous HMGs for the reduction of MOr. They studied the *k*_obs_ value under various contents of NaBH_4_. They reported that the *k*_obs_ value increased from 0.243 to 0.268 min^−1^ on increasing the NaBH_4_ from 1.26 to 3.84 mM while the *k*_obs_ decreased from this value to 0.240 min^−1^ on increasing the 5.46 mM of NaBH_4_. Initially, more H_2_ molecules reach the NPs surface with increasing NaBH_4_ content, owing to the availability of active sites. Upon increasing the NaBH_4_ content beyond 3.84 mM, the availability of active sites decreases to the MOr molecules owing to covering with H_2_ molecules. So, the *k*_obs_ value starts to decrease and this study investigated in UV-Vis spectroscopy.

The temperature also directly influences the catalytic performance of HMGs for different organic reactions. The temperature of the medium affects the kinetic energy of the reactants, and the kinetic energy controls the speed of the approach of reactants to NPs through the diffusion rate, as given in Table 1. In this way, the approach of the reactants increased with increasing temperature. Meanwhile, some hybrid microgels are temperature-sensitive and show deswelling behavior on increasing the temperature. In this case, the diffusion rate decreases with increasing temperature after some value. The temperature after which the volume of HMGs rapidly decreases is called the volume phase transition temperature (VPTT). The *k*_obs_ value decreases upon increasing the temperature after VPTT (owing to deswelling state), and increased before VPTT (owing to swelling state) with increasing temperature. Rahman *et al.*^97^ have reported the temperature effect on the *k*_obs_ value during the reduction of nitroarenes with Ag–P(MAA). They noted that the *k*_obs_ value increased from 0.038 to 0.42 min^−1^ on shifting the temperature from 303 to 340 K. In these HMGs, there are no temperature-sensitive monomers. Therefore, the *k*_obs_ value continuously increased with temperature increases because of the increasing kinetic energy of reactants molecules. This increasing kinetic energy increases the diffusion of reactants, and hence the rapidly converted into the products. Similarly, Arif *et al.*^68^ reduced 4NAn with temperature-sensitive Pd–P(Si@CS–NIPAM–MAA) HMGs. They observed that the *k*_obs_ value increased at the starting of the temperature increases up to VPTT, and then starts to decrease on further increasing the temperature. The *k*_obs_ value of 0.57 min^−1^ was obtained at 293 K, which was increased to 0.95 min^−1^ at 305 K, owing to increasing the kinetic energy of reactants. After this temperature, the HMGs shift to a deswelling state, resulting in a lower diffusion rate. Therefore, *k*_obs_ values decreased up to 0.75 min^−1^ on further increasing to 313 K. This catalytic performance under various temperatures is also observed *via* UV-Vis spectroscopy. They also observed the *k*_obs_ value in different content of HMGs, NaBH_4_, and 4NAn and obtained values are given in Table 1.

### 4.4. Thermodynamic aspects

The *k*_obs_ value of reduction increased with increasing medium temperature. The medium temperature is directly proportional to the kinetic energy (*E*_k_) of the reactants. Therefore, the *E*_k_ of the reactants increases with increasing medium temperature. Hence, the reactants rapidly reach the NPs surface, resulting in an increase in the *k*_obs_ value. This is called Arrhenius behavior. Meanwhile, the *k*_obs_ value with the temperature-sensitive HMGs decreases with increasing the temperature after VPTT value. This behavior is called non-Arrhenius. The activation energy (*E*_a_) and pre-exponential factor (*A*) values are obtained by plotting a graph between the 1/*T* and natural logarithm of *k*_obs_ values. Eqn (13) is used for this purpose.13
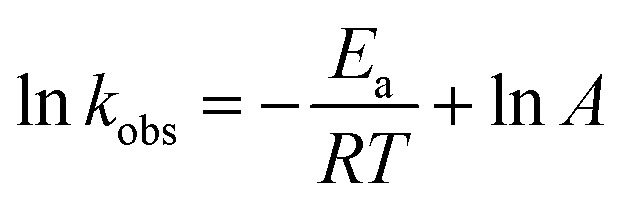


The *A* and *E*_a_ values are found from the intercept and slope of the plot ln *k*_obs_*vs.* 1/*T*, respectively. Sabadasch *et al.*^98^ have investigated the effect of medium temperature on 4NPh reduction using Pd NP-based homogeneous and core–shell HMGs, and UV-Vis spectroscopy was employed to calculate the Arrhenius components. The *E*_a_ value for 4NPh reduction was found to be 42.3 and 37.8 kJ mol^−1^ from the homogeneous and core–shell HMGs, respectively. The linear relationship between ln *k*_obs_ and 1/*T* signifies that the 4NPh reduction adheres to the Arrhenius eq. Ajmal *et al.*^99^ examined the catalytic performance of Ag–P(AA) HMGs against 4NPh, and calculated the activation entropy (ΔS*) and activation enthalpy (ΔH*) from the Eyring plot intercept and slope, respectively. Eqn (14) was used to calculate these values.14
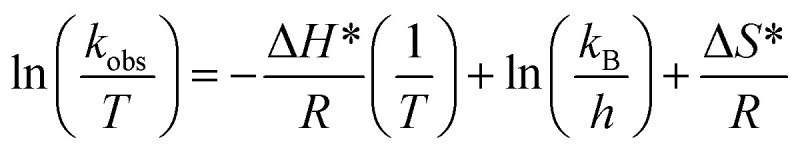


The ΔS* and ΔH* were determined to be −227.37 J mol^−1^ K^−1^ and 18.97 kJ mol^−1^, respectively. This study was also done with UV-Vis spectroscopy. Similar observations have been reported with various researchers, who monitored the reactions with UV-Vis spectroscopy to find these values.^91,95,100^

## 5. Conclusion and future directions

UV-Vis spectroscopy is an easy to use, very informative, and widely accessible technique for studying several fundamental characteristics and applications like the adsorption and catalysis of both hybrid microgels and pure systems. UV-Vis spectroscopy alone does not fully characterize the hybrid microgel systems. However, it can provide valuable information when used in conjunction with other characterization methods. For example, the volume phase transition temperature (VPTT) of thermo-responsive pure microgels is determined using the turbidity method, which involves plotting the percentage transmittance against temperature. On the other hand, it can be measured on the basis of the surface plasmon resonance wavelength (*λ*_SPR_) at varying temperatures with a UV-Vis spectrophotometer, and it is a useful approach to determine the VPTT of Au and Ag-based hybrid microgels. This method has been occasionally reported in the literature. Additionally, the stability of the plasmonic nanoparticles in microgels is assessed by monitoring their *λ*_SPR_ values over time during the storage of the dispersion medium of the hybrid microgels in the dark. There are only a few reports available that focus on measuring the transmittance of microgel dispersions to investigate the deswelling/swelling kinetics of both hybrid microgels and pure systems. A more detailed examination of the time-dependent deswelling/swelling kinetics of numerous microgels/hybrids with UV-Vis spectroscopy is required in the near future. The time required to attain the deswelling/swelling equilibrium is a crucial factor for applied purposes, and UV-Vis spectroscopy serves as an outstanding technique for this analysis. Research on the volume phase transition (VPT) of smart microgels containing plasmonic nanoparticles, assessed through *λ*_SPR_ measurements, is limited in the literature. Future studies may explore the determination of the VPTT of hybrid microgels using *λ*_SPR_ measurements.

The adsorption property of both microgels and hybrids are frequently reported for pollutants, but less commonly reported for the loading and release of drugs. The drugs are typically optically active, and their peaks lie in the UV-visible region. Therefore, the loading and release of drugs on microgels can be monitored with increasing and decreasing peak intensity in UV-Vis spectroscopy. Drug delivery is very important in the medical field. Therefore, more research is needed in the near future. The stimuli-responsive behavior (deswelling/swelling) of hybrid and pure microgels makes them perfect for drug delivery, as well as the adsorption of pollutants. Therefore, more research is required on the use of microgels and hybrid microgels for drug delivery. In addition, hybrid microgels have been used as catalysts against some pollutants. The transformation of various nitroarenes into aryl amines, reduction of different dyes, and catalytic transformation of other organic molecules are required in the near future, along with their catalytic affecting factors like the temperature, pH, and ionic strength.

## Abbreviations

AA: Acrylic acid
NIPAM: *N*-Isopropylacrylamide
NPs: Nanoparticles
MAA: Methacrylic acid
NIPMAM: *N*-Isopropylmethacrylamide
DMAAm: *N*,*N*′-Dimethylacrylamide
SMGs: Smart microgels
HMGs: Hybrid microgels
CS: Chitosan
MOr: Methyl orange
MRe: Methyl red
CRe: Congo red
FTIR: Fourier transformed infrared spectroscopy
MBl: Methylene blue
TEM: Transmission electron microscopy
RDB: Rhodamine-B
EY: Eosin Y
4NPh: 4-Nitrophenol
3NPh: 3-Nitrophenol
NMBA: *N*,*N*′-Methylene-bisacrylamide
2NPh: 2-Nitrophenol
AMPS: 2-Acrylamido-2-methylpropane sulphonic acid
HEMA: 2-Hydroxyethyl methacrylate
4NAn: 4-Nitroaniline
3NAn: 3-Nitroaniline
SEM: Scanning electron microscopy
2NAn: 2-Nitroaniline
*E*_k_
: Kinetic energy
VCLa: *N*-Vinylcaprolactam
4CNBe: 4-Chloronitrobenzene
2CNBe: 2-Chloronitrobenzene
SPR: Surface plasmon resonance
UV-Vis: UV-visible spectroscopy
BLi: Brilliant
VPTT: Volume phase transition temperature
EBT: Eriochrome black T
XPS: X-ray photoelectron spectroscopy
AFM: Atomic force microscopy
XRD: X-ray diffraction
DLS: Dynamic light scattering

## Data availability

Data will be provided on request.

## Conflicts of interest

There is no conflict of interest.
